# *Onosma mutabilis*: Phytochemical composition, antioxidant, cytotoxicity, and acute oral toxicity

**DOI:** 10.1002/fsn3.2544

**Published:** 2021-08-28

**Authors:** Ahmed Aj. Jabbar

**Affiliations:** ^1^ Department of Medical Laboratory Technology Erbil Technical Health College Erbil Polytechnic University Erbil Iraq

**Keywords:** antioxidant, cytotoxicity, *Onosma mutabilis*, oral toxicity, phytochemistry

## Abstract

The traditional use of Onosma L. species as a remedy motivated scientists to discover great biological/pharmacological potentials in this plant. In the current study, in addition to the phytochemical composition of methanol (MeOH), water, and ethyl acetate extract of aerial parts of *Onosma mutabilis* Boiss., an endemic plant species in the flora of Kurdistan, Iraq, in vitro antioxidant, cytotoxicity, and oral toxicity activity were investigated. Results of total phenolic and total flavonoid tests show the MeOH extract superiority, and the results of Gas chromatography–mass spectrophotometer(GS/GS‐MS) show 18 chemical compounds in the MeOH extract, and the majority of the detected compounds were alkaloids (78.77%) and steroids (11.48%), namely as 5,8‐dihydroxy‐2‐(4‐methylpent‐3‐enyl) naphthalene‐1,4‐dione (48.60%), 3‐O‐Methyl‐d‐glucose (27.49%), β‐Sitosterol (6.81%), Phenol, 2,4‐bis (1,1‐dimethyl ethyl)‐, phosphite (3.46%), and 24,25‐Dihydroxycholecalciferol (3.14%). Results of the antioxidant tests show the MeOH extract superiority in the phosphomolybdenum assay, radical scavenging [on 1,1‐diphenyl‐2‐picrylhydrazyl (DPPH) and 2,2′‐azino‐bis (3‐ethylbenzothiazoline‐6‐sulfonic acid) (ABTS)] assays, and reducing power [cupric reducing antioxidant capacity (CUPRAC) and ferric reducing antioxidant power (FRAP)] assays (1.45, 3.54, 2.33, 1.12, 1.62, mg/ml, respectively). The cytotoxicity results of the plant extract are presented as IC_50_ (inhibitory concentration at 50%) on the prostate cancer cells (DU‐145), mammary cancer cells (MCF‐7), and human cervix carcinoma (Hep2c), at which values ranged from 28.79 to 41.83 μg/ml. Results of the acute toxicity in the dose‐dependent trail (100, 200, 300, 600 mg/kg of MeOH) show the absence of the behavior and appearance changes of female Wister rats. Overall, *O. mutabilis* extract exhibited significant natural potentials probably because of its polar phytochemicals, which could be an alternative source for remedial, nutrient, and cosmetic manufacture.

## Graphical abstract

*Onosma mutabilis* is an endemic plant to flora of Kurdistan, Iraq, and current study aimed to investigate phytochemical profile, antioxidant, cytotoxicity, and oral toxicity activity of MeOH extract of *O. mutabilis*. Results showed increased amount of alkaloids (78.77%) and steroids (11.48%) in the extract, and biological tests showed significant antioxidant and antitumor activity of *O. mutabilis* extract against prostate, breast, and human cervix carcinoma cancer cell lines.

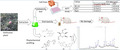

## 1 INTRODUCTION

The plant is one of the 150 Onosma species known to exist in the Asia continents. The Onosma L. genus belongs to the family Boraginaceae that has been known to exist in the tropical, subtropical, and temperate areas of the world. Turkey is the richest Asian country in terms of Onosma species (95 species) followed by China and Pakistan with 29 and 8 species, respectively. Although earlier reports show about 200 species worldwide, continued systematic botanical studies increased the number of Onosma species, and it is estimated now to reach approximately 230 species (Özgen et al., 2004; El‐Shazly et al., 2003; Kumar et al., 2013). The medicinal and industrial importance of Onosma species attracted researchers to explore more about their phytochemical and biological activities in recent years (Pal & Chaudhury, 2010).

Natural products have gained more attention in recent years because of the drawback related to chemically synthetic drugs. Growing plants rich in certain phytochemicals provide new scope to the cosmetic pharma and agro‐industry. A natural metabolically inactive glucose analog called 3‐O‐Methyl‐D‐Glucose offers significant protection for dried mouse sperm at above freezing temperatures without the need for poration of the cell membrane and improves desiccation tolerance of keratinocytes (Liu et al., 2012); (Norris et al., 2006). Furthermore, 3‐O‐Methyl‐d‐glucose (methyl glucose) is often used to study blood–brain barrier transport and the distribution spaces of hexoses in the brain (Jay et al., 1990). Continued exploration of the genus Onosma species revealed important phytochemicals including flavonoids (hesperidin, apigenin, luteolin), phenolics (rosmarinic acid, ferulic acid, vanillic acid), alkannin, and shikanon also known as isohexenyl‐naphthazarins in *O. pulchra*, *O. ambigens*, *O. gigantea*, and *O. heterophyllum*, each possessing several biological and pharmacological activities (Ozer et al., 2018; Sarikurkcu et al., 2018, 2020a, 2020b). The researchers also suggested *O. bracteatum* as a drug candidate for the cure of prostate cancer, lung cancer, and breast cancer, as a result of its cytotoxic effect against different cancer cells (Imran et al., 2018). The previous study also shown *O. arenaria* as rich source of different alkaloid classes (El‐Shazly et al., 2003). Alkaloids are organic compounds with significant biological and pharmaceutical potentials (Huizing & Malingré, 1981); (Souza et al., 2020).

The search for plant phytochemicals with greater antioxidant activity has doubled in the last decades. This rising interest in this biological activity of the plant is started when people start to realize the harmful effect of food preserves, which are used to delay exploiting of food, on human health (Menghani et al., 2011). The health damage of food additives was mainly linked to metabolic disorders such as gastrointestinal disorders, cancer, and arthritis, which results from oxidative stress, a condition when the antioxidant defense system cannot neutralize the number of reactive oxygen species and free radicals that are produced by or enter the body. Antioxidant defense system includes innate biological produced elements such as superoxide dismutase, catalase, and hydro peroxidase, and acquired antioxidant molecules from plants. Therefore, consuming plants enriched with antioxidant molecules will empower the antioxidant defense system in fighting free radicals and minimize oxidative damage (Shanmugam et al., 2018). Plant antioxidants mainly include phenolics, flavonoids, and alkaloids, and choosing a suitable extraction method necessary to maximize gained plant antioxidants because of phytochemical or solvent polarity (Wangensteen et al., 2004).

Cancer is a major health problem that includes almost 200 types of cell lines, resulting in uncontrolled cell proliferation and differentiation as spreading into surrounding tissues and organs. The curing process of the cancer patients usually detained by many obstacles, mainly drug resistance, toxicity, and decreased specification (Vukic et al., 2017). Thus, the search for new natural curative agent for controlling those health problems has been doubled in recent years. Naphthoquinones from *O. Visianii Clem* and *O. paniculata* were reported as a strong antiproliferative compound against breast carcinoma, colon cancer, and melanoma cell lines (Kretschmer et al., 2012; Vukic et al., 2018). Furthermore, the phenolic compounds from *O. aucheriana* showed a significant cytotoxicity effect against rhabdomyosarcoma, human cervix carcinoma, and murine fibroblast cell lines (Mašković et al., 2015).

The medicinal plant's increasing demand by consumers led to the development of multiform supplements from a single plant by various methods. As a result, natural compound studies majorly focused on the identification and maximizing gained isolates, ignoring the chemical profile changes that could threaten a consumer's health. (Sellami et al., 2011; Wojdyło et al., 2014). Hence, toxicity test is considered as a valuable mandatory safety measure for any medicinal plant that claimed traditionally to have curative effects (Newman & Cragg, 2012; Pariyani et al., 2015). The current work inspiration is taken from the traditional usage of onsoma species and considered as the first record on the chemical profile and biological activity of *O. mutabilis*.

## 2 MATERIALS AND METHODS

### 2.1 Plant collection

**FIGURE 1:**
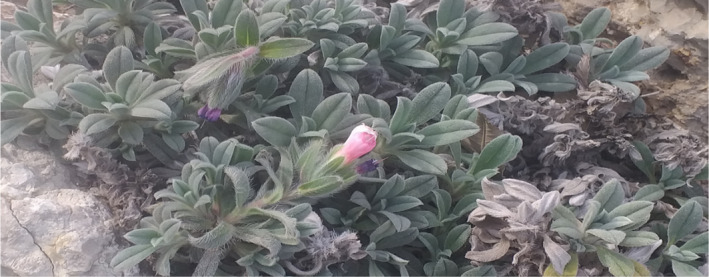
The general appearance of *Onosma Mutabilis* in the nature

The aerial part of *O. Mutabilis* was collected from Hiran/shaqlawa, Erbil, Iraq, on March 22, 2021 (Altitude; 36.40919, Longitude; 44.23401) (Figure 1). The plant identification was performed by Prof. Dr. Abdullah Shakur Sardar and deposited from the Salahaddin University Herbarium‐Education College (ESUH) (voucher no. 7852).

### 2.2 Sample preparation

The plant was dried in a shaded place at room temperature for 14 days. The ultrasound‐assisted extraction (UAE) was applied by using an ultrasonic water bath (B‐220, Branson and SmithKline Company, Danbury, CT, USA). Extract preparation were made by macerating air‐dried sample (100 g) taken from aerial parts of *O. mutabilis* with 1 L of methanol (99.9% absolute methanol), water, and ethyl acetate extracting solvents by aluminum foil and incubated in an ultrasonic bath at room temperature for two hours. The solvents drained out by a rotary evaporator in a water bath at 40℃ to obtain the solid crude extract; then, freeze‐drying was performed to complete solvent removal. The obtained dry extract was 23.67, 9.42, and 2.34% (w/w) for the MeOH, water, and ethyl acetate extracts, respectively. Then, they were stored at +4℃ until analyzed (Vardanega et al., 2014).

### 2.3 Phytochemical analysis

The MeOH, water, and ethyl acetate extract of *O. mutabilis* were investigated qualitatively for total phenolic and total flavonoid by using the spectrophotometer technique (Kähkönen et al., 1999). Then, the MeOH extract was examined qualitatively by using the GC/GC‐MS technique (Vardanega et al., 2014); (McLafferty et al., 1989). The analysis detail of both techniques is given in the Appendix S1.

### 2.4 Determination of biological activity

The MeOH, water, and ethyl acetate extract of *O. mutabilis* were investigated for antioxidant activity by using different assays: phosphomolybdenum, DPPH, ABTS, FRAP, and CUPRAC assays (Zengin et al., 2015). In addition, cytotoxicity activity of the MeOH extract of *O. mutabilis* was evaluated against the growth of malignantly transformed cell lines (prostate cancer cells (DU‐145), mammary cancer cells (MCF‐7), Hep2c (cell line derived from human cervix carcinoma—HeLa derivative) by MTT (3‐[4,5‐dimethylthiazol‐2‐yl] 2,5diphenyltetrazolium bromide) assay (Mosmann, 1983; Rana et al., 2014). And finally, the subacute toxicity test performed on female Wister rats for 7 days (Vardanega et al., 2014). All the details of the performed tests are available in the Appendix S1.

### 2.5 Statistical analysis

The statistical analysis of the biological activity was applied in triplicate to ensure the accuracy, and results are presented as mean and standard deviation (mean ± *SD*). The differences in the test activity between the extracts were found by Student's *t* test (*α* = 0.05) using SPSS v. 14.0 program. The obtained data were analyzed by using one‐way analysis (ANOVA) of the SPSS statistical software package, version 24.0 for Windows. The values of the significance were set at *p* < .05.

## 3 RESULTS AND DISCUSSION

In the current study, phytochemical profile, in vitro antioxidant, cytotoxicity, and oral toxicity activity of the MeOH extract of *O. mutabilis* were determined. In order to show the correlations between the phytochemical profile of the extract and their biological functionality, spectrophotometric and GC‐MS techniques were used to analyze the chemical constituents. The results of the chemical profile are presented in the Tables 1 and 2. The results of the antioxidant and cytotoxicity activity are presented as IC_50_ values in the Tables 3 and 4, respectively. Finally, the oral toxicity activity is presented in the Table 5. The data of the following sections are handled in a systematic order.

**TABLE 1:** Show the percentage yield, total flavonoid, and total phenolic concentration of the extracts

Assay	MeOH	H_2_O extract	Ethyl actate
Yield %	23.67^a^	9.42^b^	2.34^c^
Total Phenolic (mg REs/ g extract)	37.24^a^	21.45^b^	11.21^c^
Total flavonoid (mg GAEs/ g extract)	26.78^c^	15.32^b^	7.86^c^

Note

‐Different subscripts within the same rows show comparison between different parts by Tukey's test at *p* < .01.GAEs, REs, gallic acid, and rutin equivalents, respectively.

**TABLE 2:** Show the phytochemical contents of *O. mutabilis* analyzed by GC‐MS

No	RT (min)^a^	(Area%)^b^	Name	Molecular formula	Molecular weight g/mol	(Ref)^c^
1	17.58	0.39	2‐(5‐acetyl‐2‐furyl)‐1,4‐naphthoquinone	C_16_H_10_O_4_	266.25	1
2	17.87	0.41	Dodecane, 3‐methyl‐	C_13_H_28_	184.36	2
3	22.343	0.39	Pentacosane	C_25_H_52_	352.69	3
4	24.563	0.99	Cyclopentane, 1,2‐dimethyl‐3‐(1‐methylethenyl)‐	C_10_H_18_	138.24	4
5	25.264	0.45	2‐Octyne	C_8_H_14_	110.19	3
6	27.443	48.60	5,8‐dihydroxy‐2‐4‐methylpent‐3‐enyl,naphthalene‐1,4‐dione	C_16_H_16_O_5_	288.29	5
7	29.43	0.48	Octadecane	C_18_H_38_	254.49	3
8	34.069	0.93	n‐Dodecyl glycidyl ether	C_15_H_30_O_2_	242.40	6
9	34.386	1.73	Diisooctyl phthalate	C_24_H_38_O_4_	390.6	7
10	35.506	27.49	3‐O‐Methyl‐d‐glucose	C_7_H_14_O_6_	194.18	8
11	36.575	0.90	1‐Nonadecene	C_19_H_38_	266.5	3
12	39.014	1.02	Eicosane	C_20_H_42_	282.5	3
13	40.783	0.56	2,4,6‐TRIMETHYLMORPHOLINE	C_7_H_15_NO	129.2	9
14	41.224	3.14	24,25‐Dihydroxycholecalciferol	C_27_H_44_O_3_	416.6	10
15	42.656	1.53	delta.5‐Ergostenol	C_28_H_44_O	396.65	10
16	43.974	6.81	β‐Sitosterol	C_29_H_50_O	414.71	11
17	44.566	0.73	1‐Heptatriacotanol	C_37_H_76_O	537	12
18	45.645	3.46	Phenol, 2,4‐bis(1,1‐dimethylethyl)‐, phosphite	C_42_H_63_O_3_P	646.92	13

Compound type (Total number)	% in MeOH extract Aerial part
Alkaloids (5)	78.77%
Steroid (3)	11.48%
Hydrocarbons (9)	9.02%
Alcohol (1)	0.73%
Total (18)	%100

^a^ Retention time (tR [min]) on a Restek Rtx‐5 column.

^b^ Peak area percentage calculated from the GC‐FID chromatogram. NF: compound not found.

^c^ References used to classify the nature of compound ^1^(Molleti & Singh, 2015), ^2^(Luning Prak et al., 2020), ^3^(Schmidt et al., 2014), ^4^(Prakasia & Nair, 2015), ^5^(Vukic et al., 2017), ^6^(Lim et al., 2015), ^7^(Autian, 1973), ^8^(Norris et al., 2006), ^9^(Souza et al., 2020), ^10^(Bikle, 2014), ^11^(Babu & Jayaraman, 2020), ^12^(Junwei et al., 2018), ^13^(Lee, 2010).

**TABLE 3:** Antioxidant activity of *O. mutabilis* ^1^ by using different assays

Assay	phosphomolybdenum assay^2^	DPPH scavenging^2^	ABTS scavenging^2^	FRAP reducing^3^	CUPRAC reducing^3^
Methanol	1.45 ± 0.05^b^	3.54 ± 0.064^a^	2.33 ± 0.045^a^	1.12 ± 0.023^b^	1.62 ± 0.079^a^
Water	2.14 ± 0.09^c^	4.27 ± 0.021^a^	3.61 ± 0.23^a^	1.34 ± 0.08^c^	1.86 ± 0.03^a^
Ethyl acetate	3.91 ± 0.26^d^	54.23 ± 4.33^b^	24.86 ± 0.78^b^	2.75 ± 0.05^d^	3.06 ± 0.09^b^
Trolox	1.03 ± 0.02^a^	0.25 ± 0.02^a^	0.29 ± 0.02^a^	0.1 ± 0.01^a^	0.28 ± 0.02^a^
EDTA^5^	NF^6^	NF	NF	NF	NF

^1^ The values indicated by different superscripts within the same column are not different according to the Tukey's honestly significant difference post hoc test at 5% significance level.

^2^ IC_50_ (mg/ml), inhibition concentration at which 50% of the DPPH (2,2‐Diphenyl‐1‐picrylhydrazyl) and ABTS (2,2′‐azino‐bis‐3‐ethylbenzthiazoline‐6‐sulphonic acid) radicals were scavenged and the ferrous ion–ferrozine complex were inhibited.

^3^ EC50 (mg/ml): Effective concentration at which the absorbance was 0.5 for CUPRAC (Cupric ion reducing antioxidant capacity) and FRAP (Ferric reducing antioxidant power) assays.

^5^ EDTA: Ethylenediaminetetraacetic acid (disodium salt).

^6^ nf: Not found.

**TABLE 4:** The antiproliferative activity IC_50_ (μg/ml) of *O. mutabilis* extract on the DU‐145, MCF‐7, and Hep2c after 24 h of treatment

Cell line	IC50 values (μg/ml)^1^	
	*O. mutabilis* MeOH extract	DOX^2^
DU‐145	35.67 ± 0.15	0.87 ± 0.48
MCF‐7	28.79 ± 0.23	0.67 ± 0.34
Hep2c	41.83 ± 0.21	1.27 ± 0.12

Note

Key; DU‐145; prostate cancer cells, MCF‐7; mammary cancer cells, Hep2c; human cervix carcinoma.

^1^ Mean value ± *SD* of IC_50_ (μg/ml), inhibition concentration at which 50%.

^2^ DOX; Doxorubicin.

**TABLE 5:** Effect of different dosage of the methanolic extract of *O. mutabilis* on the behavior, appearance, and life status of rats

Parameters	G1	Rates fed on MeOH extract mg/kg			
		G2	G3	G4	G5
Feed and water intake	N	N	N	N	N
Coma	A	A	A	A	A
Convulsion and tremors	A	A	A	A	A
Eyes	N	N	N	N	N
Faces consistency	N	N	N	N	N
Fur and skin	N	N	N	N	N
Itching	A	A	A	A	A
Respiration	N	N	N	N	N
Sleep	N	N	N	N	N
Urination (color)	N	N	N	N	N
Aggressiveness	A	A	A	A	A
Mortality	A	A	A	A	A

Note

Key. A—Absent; P—Present; N—Normal; ↑—Increase. G1—Control rats with no supplementation; G2, G3, G4, and G5 are female rats receiving 100, 200, 300, and 600 mg/kg of methanolic extracts of *O. mutabilis*, respectively.

### 3.1 Phytochemical profile

As declared by now, the current work investigates the chemical profile and the biological activity of *O. mutabilis* extract. Thus, the chemical profile results are discussed at first. The chemical constituents analyzed by two different methods, qualitative method applied for the total phenolic and total flavonoid estimation by the spectrophotometer (Table 1). It was found that the MeOH extract contained a higher amount of the phenolic and flavonoid compounds than that for the water and ethyl acetate extracts, respectively. The determined amount of the phenolic and flavonoid compounds of the MeOH extract was 37.24 mg GAEs/g extract and 26.78 mg QEs/g extract, respectively. The phenolic and flavonoid contents of the water extract was found as 21.45 mg GAEs/g extract and 15.32 mg GAEs/g extract, respectively. While the phenolic and flavonoid contents of the ethyl acetate extract were 11.21 GAEs/g extract and 7.86 mg QEs/g extract, respectively. As expected, ethyl acetate was the poorest in terms of total phenolic and flavonoid contents.

**FIGURE 2:**
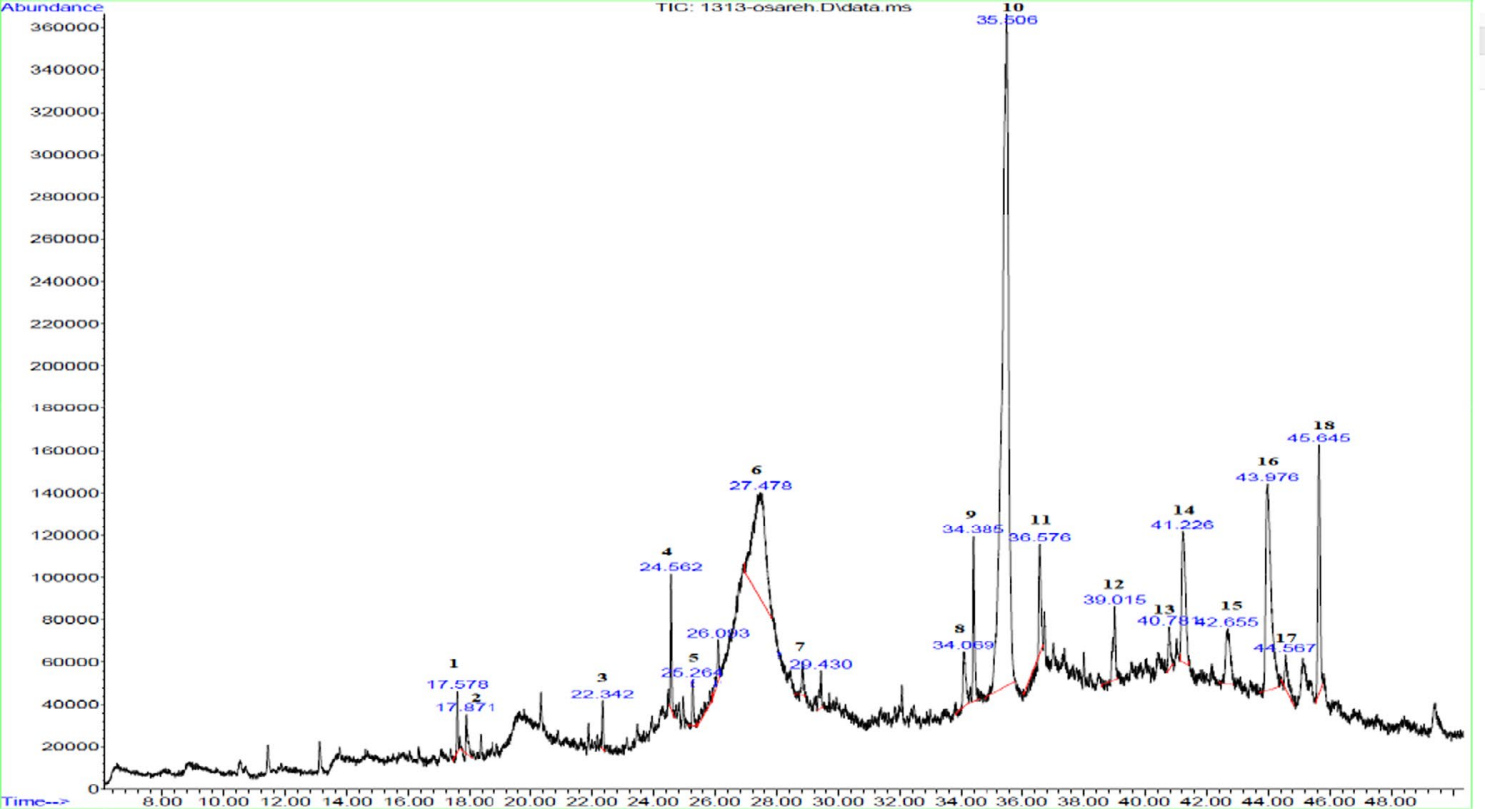
Chromatogram of methanolic extract Of *O. Mutabilis*. 1‐18 represent chromatogram of 2‐(5‐Acetyl‐2‐Furyl)‐1,4‐Naphthoquinone, Dodecane, 3‐Methyl‐, Pentacosane, Cyclopentane, 1,2‐Dimethyl‐3‐(1‐Methylethenyl)‐, 2‐Octyne, 5,8‐dihydroxy‐2‐4‐methylpent‐3‐enyl,naphthalene‐1,4‐dione (Deoxy shikonon), Octadecane, N‐Dodecyl Glycidyl Ether, Diisooctyl Phthalate, 3‐O‐Methyl‐D‐Glucose, 1‐Nonadecene, Eicosane, 2,4,6‐Trimethylmorpholine, 24,25‐Dihydroxycholecalciferol, Delta.5‐Ergostenol, Β‐Sitosterol, 1‐Heptatriacotanol, Phenol, 2,4‐bis(1,1‐dimethylethyl)‐, phosphite

Second, to analyze the chemical profile of *O. mutabilis* in detail, a GC‐MS technique is applied only for the MeOH extract since it was the richest extract in all studied parameters and an amount of eighteen standard compounds was found, which constitute about 100% of the methanolic extract of *O. mutabilis* (Table 2). In the MeOH extract, despite some small amount of phytochemicals, it was understood the main constituents of the extract are 5,8‐dihydroxy‐2‐(4‐methylpent‐3‐enyl) naphthalene‐1,4‐dione (Deoxy shikonin) (48.60%), 3‐O‐Methyl‐D‐Glucose (27.49%), Β‐Sitosterol (6.81%), Phenol, 2,4‐bis (1,1‐dimethyl ethyl)‐, phosphite (3.45%), and 24,25‐Dihydroxycholecalciferol (3.14%), respectively. Although they are present in low amount, the extract contained notable amount of Diisooctyl Phthalate (1.73%), Delta.5‐Ergostenol (1.53%), Eicosane (1.01%), N‐Dodecyl Glycidyl Ether (0.93%), and Cyclopentane,1,2‐Dimethyl‐3‐(1‐Methylethenyl) (0.99%), respectively. While some compound of the extract was present in negligible amount including 1‐Nonadecene (0.90%), 1‐Heptatriacotanol (0.73%), Octadecane (0.48%), 2,4,6‐Trimethylmorpholine (0.56%), 2‐Octyne (0.45%), Dodecane,3‐Methyl‐ (0.41%), 2‐(5‐Acetyl‐2‐Furyl)‐1,4‐Naphthoquinone (0.39%), and Pentacosane (0.39%) (Table 2) (Figure 2).

The organic class of major compounds of the methanolic extract of *O. mutabilis* was alkaloids (78.77%) followed by steroids (11.48%), hydrocarbons (9.02%), and alcohol (0.73%), respectively. The previous research works declared alkaloids and naphthoquinones as the main phytochemical component of *O. erecta Sibth. & Sm., O. kaheirei, O. arenaria pennina Br.‐Bl., O. hetrophyllum, Onosma microcarpum* (Damianakos et al., 2014a; El‐Shazly & Wink, 2014; Mellidis, 1988; Wiedenfeld & Abbildungen, 1993). In addition, a study by Vukic et al. showed seven napthoquinon (deoxyshikonin, isobutyrylshikonin, alpha methylbutyrylshikonin, acetylshikonin, alpha‐hydroxyisovalerylshikonin, 5,8‐O‐dimethyl isobutyrylshikonin, and 8‐O‐dimethyl deoxyshikonin) as the main chemical contents of *O. Visianii* extract (Vukic et al., 2017). Furthermore, chromatographical study on *O. argentatum* extract reported deoxyshikonin, acetyl shikonin, 3‐hydroxy‐isovaleryl shikonin, and 5,8‐O‐dimethyl acetyl shikonin as their main phytochemical constituent (Pavol et al., 2008). Similarly, almost the same naphthoquinone derivatives reported by an earlier phytochemical study on the *Onosma exsertum Hemsl., O. hookerii Clarke, Onosma waltonii Duthic, Onosma confertum W.W. Smith, and Onosma hookerii Clarke* var. longiflorum Duthie (Attar & Joharchi, 2006). Literature search on the chemical profile of *O. mutabilis* has not detected any previous research study elsewhere. Therefore, the presented data considered as the first record for the literature and could be the starting line for future investigations on this plant.

### 3.2 Antioxidant activity

The investigation of the antioxidant activity of *O. mutabilis* was performed by using different assays. The phosphomolybdenum assay was applied to test total antioxidant activity. The DPPH and ABTS assays were applied to test the free radical scavenging activity of the extracts. Finally, the CUPRAC and FRAP assays were applied to evaluate the reducing power activity. The data results are presented in the (Table 3).

In the phosphomolybdenum assay, the phosphatemolybdenum is incubated with the extract, and the conversion of phophatemolybdenum is estimated to reveal the total antioxidant activity of the extract. The IC_50_ value of the methanolic extract in the antioxidant activity was 1.45 ± 0.05 mg/ml and was found to be very close to the Trolox value (1.03 ± 0.02 mg/ml) (Table 3). The antioxidant activity of the water extract and the ethyl acetate extract was 2.14 ± 0.09 and 3.91 ± 0.26 mg/ml, respectively, and was found to be lower than that of the MeOH extract. The results presented here are good compatible with the chemical profile of the extracts. Based on the Tukey's test results, the total antioxidant activity of the MeOH extract and the Trolox was significantly differed at %5 (Table 3).

The radical scavenging ABTS assay showed higher scavenging activity by the extracts than that from the DPPH assay. The MeOH extract showed to be more influential in the scavenging DPPH and ABTS radicals that could be linked with its higher phenolic and flavonoid contents (Table 3). The MeOH extract showed higher radical scavenging activity on DPPH and ABTS assays as results show 3.54 mg/ml and 2.33 TEAs mg/ml, respectively. It was followed by the scavenging activity of water extract with values of 4.27 and 3.61 TEAs mg/ml on DPPH and ABTS radicals, respectively. The ethyl acetate extract showed minimum free radical scavenging activity in comparison with other extracts as expected. The free radical scavenging efficiency of the ethyl acetate extract was recorded as 54.23 and 24.86 TEAs mg/ml on DPPH and ABTS radicals, respectively.

The reducing power activity is presented by electron‐based technique (CUPRAC), and estimation of the capacity of the extracts to reduce Fe3+ ions (FRAP) (Table 3). All the extracts showed higher reducing capacity on the FRAP assay than that of the CUPRAC assay. In the FRAP assay, the reducing ion capacity of the methanolic extract was estimated as 1.12 TEs mg/ml extract, a very close value to 1.34 TEs mg/ml extract of water, but significantly higher than that of 2.75 TEs mg/ml extract for the ethyl acetate extract. While in CUPRAC assay, the reducing ion capacity of the MeOH extract was measured as 1.62 TEs mg/ml extract, a very close value to 1.86 TEs mg/ml of the water extract, but significantly higher than that of 3.06 TEs mg/ml extract for the ethyl acetate extract. The reducing ion capacity of the positive standard was higher than that of the plant extracts. In both assays, the obtained data for the MeOH and water extracts were not statistically different. However, significantly lower values were given by both assays for the ethyl acetate extract in comparison with other extracts.

According to my literature search, reports on the antioxidant activity of *O. mutubilis* have not been published elsewhere. However, some studies have been done on the antioxidant activity of some phytochemicals that were thought to have this property. A previous antiradical study correlated deoxyshikonin, pyrrolidine, and pyrrolizidine alkaloids detected from *Anabasis articulate* to the plant's antioxidant potential (Belyagoubi‐Benhammou et al., 2019). The presence of pyrrolizidine alkaloids and their biological roles including antioxidant activity has been reported in various Onosma species including *O. arenaria*, *O. columnae*, *O. eptantha*, *O. erecta*, *O. kaheirei* (El‐Shazly et al., 2003; Yıldız et al., 2020; Damianakos et al., 2014a). The earlier in vivo investigation showed significant antioxidant activity of *Onosma armeniacum K*. extract and have correlated this activity to the plant's naphthoquinone contents (Cadirci et al., 2007). In the current study, phytochemical profiling showed alkaloids as the main constituents (78.77%) which may be responsible for their efficient antioxidant potentials. The previous study has shown a positive correlation between the increased antioxidant potential of maca extract with its high alkaloids contents 62.4% (Gan et al., 2017). The current study also detected a significant amount of β‐sitosterol, a herbal nutraceutical that are recently highlighted as antioxidant and possibly futuristic remedy for numerous health problems (Babu & Jayaraman, 2020). Furthermore, 2,4‐bis (1,1‐dimethyl ethyl)‐, phosphite (alkanox240) was found in significant amount (3.45%) that has been known as an organo‐phosphite antioxidant exhibiting excellent hydrolytic stability and reduces peroxide‐induced oxidative degradation of most polymeric substances (Johnson et al., 2005). The 24,25‐Dihydroxycholecalciferol was another significantly detected compound in the current study, a well‐known antioxidant rolling as an antiaging agent by reducing oxidative stress and DNA damages, lowering cell senescence, and deactivating p53‐p21signaling pathways (Richardson, 2001). The literature data cited above are considered as reliable confirmation on the contribution of detected phytochemicals to the antioxidant activity of *O*. *mutabilis* extracts.

### 3.3 Cytotoxicity activity

Medicinal herbs have been used therapeutically for ages and are still considered as raw materials for modern remedies. According to the previous estimation, natural products contribute to 60% of today's anticancer drug production (Gordaliza, 2007). Another study reported that herbal medicine is used as a curative agent by 50% of breast cancer and by 37% of prostate cancer patients (Richardson, 2001). Previous preclinical studies showed the efficacy of natural compounds to inhibit human cervical carcinoma and other cancers (Park et al., 2021); (Aung et al., 2017). However, most of them have not been well screened for their biological activity, especially their cytotoxicity effect. In the current study, cytotoxicity activity of *O. mutabilis* extracts was estimated by ultrasound technique on three different cell lines: cancer cells derived from prostate cancer (DU‐145), mammary cancer (MCF‐7), and human cervix carcinoma Hep2c (HeLa), as presented in the (Table 4). Moreover, doxorubicin was used as a reference against the same cancer cell lines.

Data results of IC_50_ value for the methanolic extract of *O. mutabilis* ranged from 28.79 to 41.83 μg/ml. According to my systematic search, reports on the cytotoxicity effect of *O. mutabilis* have not been published elsewhere. However, researchers have shown some phytochemicals that were thought to have antiproliferative effect. The previous study correlated the antitumor effect of *Liparis Nervosa* extract with its pyrrolizidine alkaloid contents (Chen et al., 2018). The current study showed an increased percentage of alkaloids in the methanolic extract of *O. mutabilis*, which could be correlated with its significantly cytotoxicity activity. Previously, the anticancer potentials of alkaloids from traditional medicinal plants have been well screened by highlighting the molecular mechanism of their actions (Mondal et al., 2019). The earlier study also correlated the anticancer activity of *O. nigricaule* extract with its alkaloid contents, namely deoxyshikonin, b,b‐dimethylacryl shikonin, and acetyl shikonin (Ozgen et al., 2010). Additionally, previous researchers reported triterpenoid, bauerenone, and β‐sitosterol from *Onosma limitaneum* extract as effective antiproliferative agent against various tumor cells (Ahmad et al., 2005). Furthermore, several studies have reported shikonin as anticancer agent because of its ability to induce apoptosis in different human tumor cells from gastric cancer, prostate cancer, and breast cancer (Liang et al., 2016; Gara et al., 2015; Lin et al., 2018). Different mechanisms proposed on how shikonin induce apoptosis, and a particular study detected that shikonin stimulates p53‐mediated cell cycle arrest and apoptosis in A375‐S2 melanoma cells by caspase 9‐dependent mechanism (Wu et al., 2004), while another study reported that shikonin induces apoptosis by stimulating reactive oxygen species (ROS)‐mediated endoplasmic reticulum (ER) stress and p38 pathways (Liu et al., 2019). The phytochemical profiling of *O. mutabilis* also showed a significant amount of β‐Sitosterol, a nutraceutical product that was reportedly confirmed as an anticancer agent against various tumors cells (Bin Sayeed & Ameen, 2015). Until now, clear mechanism explaining the anticancer activity of β‐sitosterol has not been reported yet, but in vivo study reported that the β‐sitosterol induced immune response and significantly lowered the amount of metastases in experimented animals injected with lung cancer cell line. This particular anticancer effect was correlated with the positive impact of β‐sitosterol on the gut immune surveillance systems (Awad et al., 2001). Furthermore, earlier study reported that β‐sitosterol detained colonic epithelial cell proliferation because of its decreased absorption property (Baskar et al., 2012). The literature referenced above was considered as the reliable data on the involvement of detected phytochemicals in the cytotoxicity activity of *O. mutabilis* extract against tested cell lines.

### 3.4 Oral toxicity test

The obtained information on the oral toxicity test showed a lack of undesired changes in the appearance, behavior, and life status of rats under treatment of 100, 200, 300, and 600 mg/kg of the MeOH extract of *O. mutabilis* for 7 days (Table 5).

Exploring the acute toxicity of various doses of the MeOH extract in the current study can be considered a useful safety control for the potentiality of consuming it in repeated administrations. Mortality, appearance, and behavioral changes are considered as the starting signs of toxicity usually noticed in acute toxicity studies (Lee et al., 2014). Interestingly, our subacute toxicity study showed no significant sign of toxicity. As the rats were safe even after 600 mg/kg administration for 7 repeated days, the predicted LD_50_ of the extract would be higher than 600 mg/kg body weight. According to the results of the literature search, reports on the toxicity effect of *O. mutabilis* have not been published elsewhere. But various toxicity studies on other Onosma species showed the safety usage of this plant in dose‐dependent trials. The previous research studies shared that feeding trials of rats become nontoxic at a low dose and toxic at higher doses of *O. hispidum* wall. Extract, a well‐known plant for Ratanjot red dye for food coloring (Khalili et al., 2010); (Redzić et al., 2009). Another in vivo toxicity study on rats showed the safety of *O. echioides* extract in subacute toxicity test and dose‐dependent increased mortality of rats in acute toxicity test with the LD_50_ claimed to be 1,000 mg/kg body weight (Shoaib et al., 2020). The data provided here can be considered as the first step toward the identification of the phytochemical and biocompatibility of *O. mutabilis*. However, further studies are required to explore the mechanism of the actions possessed by the phytochemicals and to investigate the toxicity effect of *O. mutabilis* extract at higher doses for longer periods.

## 4 CONCLUSION

In the current study, the phytochemical profile of *O. mutabilis* was provided as useful data behind its biological potentials, namely antioxidant and cytotoxicity activity. Antioxidant activity test results showed the methanolic extract as the strongest antioxidant agent that could serve as food additive to delay expiration. Phenolic, flavonoid, and alkaloid contents, especially Deoxy shikonin, β‐sitosterol, Phenol, 2,4‐bis (1,1‐dimethyl ethyl)‐, phosphite, and 24,25‐Dihydroxycholecalciferol, are thought to be involved in the antioxidant activity of the extracts. The cytotoxicity potentials significantly exhibited by the methanolic extract, which may be correlated with its alkaloid and β‐sitosterol contents. Hence, encouraging by their natural potentials, the plant species can be an alternative and valuable source for remedial, nutrient, and cosmetic manufacture.

## CONFLICT OF INTEREST

The author declares no conflict of interest.

## ETHICAL APPROVAL

The study followed the Iraqi ethical guidelines for the care and use of laboratory animals, and the study protocol was approved by the ECETHC (Ethical Committee of Erbil Technical Health College) (Reference No. 34 in 25‐06‐2021).

## Supporting information

Supplementary Material

## Data Availability

The materials related to this study are available in the Appendix S1, in the online version, at doi:
