# Supplementary material for: Onosma mutabilis: Phytochemical composition, antioxidant, cytotoxicity, and acute oral toxicity
Source: Food Sci Nutr. 2021 Aug 28;9(10):5755–64. doi: 10.1002/fsn3.2544 (PMC8498047; doi:10.1002/fsn3.2544)
Supplement: Supplementary file 1 — Supplementary Material [file FSN3-9-5755-s001.docx]

Supplementary file

**2. MATERIALS AND METHODS**

*2.1 Plant collection*

The aerial part of *O. mutabilis* was collected from Hiran/shaqlawa, Erbil-Iraq on 22 March 2021 (Altitude; 36.40919, Longitude; 44.23401) (Figure 1). The plant identification was performed by Prof. Dr. Abdullah Shakur Sardar and the plant details were deposited from the Salahaddin University Herbarium-Education College (ESUH). (voucher no. 7852).

*2.2. Sample preparation*

The Plant was dried in a shaded place at room temperature for 14 days. The ultrasound-assisted extraction (UAE) was performed in an ultrasonic water bath (B-220, Branson and SmithKline Company, Danbury, CT, USA). Extract preparation was made by macerating air- dried sample (100 g) taken from aerial parts of *O.mutabilis* with 1 L of Methanol(99.9 % absolute methanol), water, and ethyl acetate extracting solvents by aluminum foil and incubated in Ultrasonic bath at room temperature for two hours. The solvents were separated by a rotary evaporator in a water bath at 40 °C to obtain the solid crude extract, then freeze-drying was performed to complete solvent removal. The obtained dry extract was 23.67, 9.42, 2.34 % (w/w) for MeOH, water, and ethyl acetate extracts, respectively. Then, they were stored at +4°C until analyzed (Vardanega et al., 2014).

*2.3 Phytochemical analysis*

2.3.1 Spectrophotometric technique

The obtained dry extract dissolved in methanol, water, and ethyl acetate to a final volume of 10mg/ml. Then, the solutions were analyzed spectrophotometrically for the total phenolic and total flavonoids estimation. For the total phenolic estimation, 0.5 ml of Folin–Ciocalteu reagent and sodium carbonate (20%, 1.5 mL) were added to extract solution after dilution with distal water and allowed to react at room temperature for one hour. The water was used as a blank. The measurement of the absorbance was done at 750nm (Kähkönen et al., 1999). Data expressed as equivalent to Gallic acid. For the total flavonoid estimation, extract solutions (10 mg/mL, 1 mL) were mixed with 5% sodium nitrite solution (0.3 mL) for 5 min, then aluminum chloride (10%, 0.3 mL) was added and allowed to stand for 6min, Then, sodium hydroxide (1 mol/L, 1 mL) along with distal water was added to complete volume 10 ml. The water was used as a blank and the absorbance was read at 510nm (Kähkönen et al., 1999).

2.3.2 GC-MS Technique

2.3.2.1 GC-MS conditions

The methanol extract was analyzed by using Shimadzu Model QP-2010 GC coupled with MS. GC equipped with HP-5 MS (5% phenylmethyl siloxane), capillary column (30 m × 0.25 mm i.d., film thickness 0.25μm) in the temperature program 60°C (2’) to 250 °C for 10 minutes with a rate of 20 °C /min, helium flow rate 1.61ml/minute. The ion source was maintained at 250 °C with an electron energy of 70 eV. The extracted sample was added into the methanol and then 1μl injected into the column.

2.3.2.2 Identification of compounds

Based on the Wiley GC/MS Library, Adams Library, and Mass Finder Library, the unknown component was recognized based on the comparison of their mass spectrum with the spectrum of the reference components. The identification of name, structure, and molecular weight of the detected compound were eventually made. The relative percentages of the separated compounds were calculated from FID chromatograms (Vardanega et al., 2014);(McLafferty et al., 1989). The analysis results are expressed as a mean percentage as listed in Table 1.

*2.4 Determination of the antioxidant Activity*

The antioxidant activity of the MeOH extract was evaluated using different assays [on 1,1-diphenyl-2-picrylhydrazyl (DPPH) and 2,2′-azino-bis (3-ethylbenzothiazoline-6-sulfonic acid) (ABTS)] assays, reducing power [cupric reducing antioxidant capacity (CUPRAC) and ferric reducing antioxidant power (FRAP)]. The antioxidant activity was expressed as equivalents of Trolox as previously described (Zengin et al., 2015).

*2.5 Measurement of cytotoxic activity by MTT assay*

The effect of *O. mutabilis* extract on the growth of malignantly transformed cell lines was evaluated by MTT (3-[4,5-dimethylthiazol-2-yl]-2,5 diphenyltetrazolium bromide) assay.

The following cell lines were used: prostate cancer cells (DU-145), mammary cancer cells (MCF-7), Hep2c (cell line derived from human cervix carcinoma – HeLa derivative). The cells were grown (2·105 cell/mL;100 L/well) in 96-well cell culture plates (NUNC) in a nutrient medium (MEM Eagle supplemented with 5% (for Hep2c) or 10% (for DU-145 and MCF-7FCS) and grown for 24 hours at 37 °C then, sample solution (dissolving 5mg of extract in 1 ml of methanol) and standard solution (absolute methanol) diluted with nutrient medium and add up to a volume of (100 L/well). The cells lines were grown for 48 h at 37 °C in the humidified atmosphere with 100 L of pure nutrient as a positive control for each cell line. The supernatants were separated after incubation process, MTT (stock solution: 5 mg/mL in PBS) dissolved in D-MEM medium to final concentration 500 g/ml with (100 L/well) for each well, and incubation for well plates performed at 37 °C in humidified atmosphere for 4 h. The addition of 10% SDS/10 mM HCl (100 L/well) was done to halt the reaction. Absorbance was read at 580 nm by spectrophotometer (UV1100, CE ISO, YOKE, Shanghai, China) after incubation overnight at 37 °C. The viable cells were calculated from a standard curve made from cells at 580nm.

Grown cells were used as standards after cell counting, diluted standard suspensions centrifuged at 800rpm for 10min, and then mixed with MTT/D-MEM and 10% SDS/10 mM HCl solutions. The viable cell was counted by ELISA plate reader at 580 nm as an equal proportion to the intensity of light absorbance. To calculate cell survival (%), the following equation was used:

Cell survival (%) = Absorbance of a sample with cells grown in plant extract/Absorbance of control cells grown in nutrient medium ×100.

The concentration of plant extract inhibiting cell survival by 50% in comparison to the standard treated group is expressed as IC50 value. The data of the experiments are presented as the percentage of positive standard growth taking the Doxorubicin (Dox) determined in standard control wells as the 100% growth (Rana et al., 2014);(Mosmann, 1983). The experiments were performed in triplicate.

*2.6 Acute toxicity test*

2.6.1 Animals and approval from animal ethical committee

Forty rates weighing 200-300 grams of both sexes were obtained from the animal unite of College of Education, Salahaddin University, Erbil-Iraq. The rats were kept in stainless steel cages and housed at an ambient temperature of between 25- 27°c and relative humidity of about 50-55% with free access to feed (ad libitum) and water. Ethical guidelines governing the use of live animals for the conduct of experiments as stipulated by Iraqi animal right was strictly followed and the study protocol was approved by the ECETHC (Ethical Committee of Erbil Technical Health College) provided the ethical committee approval (Ref. No. 34 at 25-06-2021).

2.6.2 Seven days repeated dose of oral Acute toxicity test on rats

The current study followed the method of Lorke (Lorke, 1983). The rats were divided randomly into the control and 4 test groups with 5 rats of either sex in each group, before treatment. Before dosing, foods were cut off for the night with free water access.

Test groups (G2, G3, G4, G5) were dosed with the (100, 200, 300, 600 mg/kg of b.w.p.o.) of MeOH extracts of aerial parts of *O.mutabilis* and the control group (G1) has received vehicle (1%Tween 80 in water, p.o.). The food was provided after 1-2 hours of dosing. The observation of animals begins immediately for the first 30 minutes and then following the oral dose. The record continued for 7 days every 8 hours. Clinical symptoms of toxicity, such as intake of food and water, convulsion, the overall behavior, and death of treated animals, were recorded (Vardanega et al., 2014).
